# Prenatal Exposure to Dexamethasone in the Mouse Alters Cardiac Growth Patterns and Increases Pulse Pressure in Aged Male Offspring

**DOI:** 10.1371/journal.pone.0069149

**Published:** 2013-07-25

**Authors:** Lee O'Sullivan, James S. M. Cuffe, Tamara M. Paravicini, Sally Campbell, Hayley Dickinson, Reetu R. Singh, Oksan Gezmish, M. Jane Black, Karen M. Moritz

**Affiliations:** 1 School of Biomedical Sciences, The University of Queensland, St. Lucia, Queensland, Australia; 2 The Ritchie Centre, Monash Institute of Medical Research, Clayton, Victoria, Australia; 3 Department of Anatomy and Developmental Biology, Monash University, Clayton, Victoria, Australia; The University of Manchester, United Kingdom

## Abstract

Exposure to synthetic glucocorticoids during development can result in later cardiovascular and renal disease in sheep and rats. Although prenatal glucocorticoid exposure is associated with impaired renal development, less is known about effects on the developing heart. This study aimed to examine the effects of a short-term exposure to dexamethasone (60 hours from embryonic day 12.5) on the developing mouse heart, and cardiovascular function in adult male offspring. Dexamethasone (DEX) exposed fetuses were growth restricted compared to saline treated controls (SAL) at E14.5, but there was no difference between groups at E17.5. Heart weights of the DEX fetuses also tended to be smaller at E14.5, but not different at E17.5. Cardiac AT_1a_R, Bax, and IGF-1 mRNA expression was significantly increased by DEX compared to SAL at E17.5. In 12-month-old offspring DEX exposure caused an increase in basal blood pressure of ∼3 mmHg. In addition, DEX exposed mice had a widened pulse pressure compared to SAL. DEX exposed males at 12 months had an approximate 25% reduction in nephron number compared to SAL, but no difference in cardiomyocyte number. Exposure to DEX *in utero* appears to adversely impact on nephrogenesis and heart growth but is not associated with a cardiomyocyte deficit in male mice in adulthood, possibly due to compensatory growth of the myocardium following the initial insult. However, the widened pulse pressure may be indicative of altered vascular compliance.

## Introduction

The developing fetus has been shown to be susceptible to perturbations in the intrauterine environment, with an increased risk of developing a number of adult-onset diseases such as hypertension, cardiovascular disease and kidney disease [1], [2]. Although the mechanisms that lead from altered fetal growth and development to adult disease are still unclear, the developing heart and kidney have been shown to be particularly susceptible to prenatal and perinatal insult [3], [4], [5]. A variety of different programming models have illustrated this susceptibility. Maternal protein restriction in rats leads to a decrease in the number of cardiomyocytes at birth, a larger heart volume at 4 weeks of age [3], [6], and a reduction in nephron number [7]. Whilst the offspring of protein restricted dams have been shown to develop increased systolic blood pressure [8], this is not universal [9], and this may be dependent on consequent perturbations in the renin-angiotensin system [10] and/or the postnatal growth trajectory [11]. Uteroplacental insufficiency in rats causes a reduction in cardiomyocyte and nephron number, vascular dysfunction and arterial stiffness: evidence of a hypertensive phenotype appears to develop only in male offspring suggesting sex specific effects in the subsequent programming of disease [12], [13], [14].

Glucocorticoids are a commonly used therapy to treat a wide variety of inflammatory conditions including asthma, and continue to be prescribed during pregnancy [15]. Elevations in glucocorticoids may be a common mechanism through which many programming insults bring about their disease phenotypes [16]. For example the administration of metyrapone, which decreases glucocorticoid synthesis, has been reported to ameliorate the increase in systolic blood pressure seen in rat offspring following maternal protein restriction during pregnancy [17]. The role of synthetic glucocorticoids such as dexamethasone (DEX) in the programming of adult-onset diseases has been well studied [4], [18], [19], [20]. These studies have consistently demonstrated a reduction in nephron endowment following DEX in sheep (0.48 mg/h, day 26–28 of pregnancy) [21], the spiny mouse (125 µg/kg day 20–23) [22] and in the rat (0.2 mg/kg on E15 and E16 or E17 and E18) [23]. In the sheep and rat studies, DEX exposure is associated with increased mean arterial pressure and impaired cardiac function [24], [25], [26]. In the spiny mouse, offspring do not have an increase in basal blood pressure [22]. Interestingly, in the rat when O'Regan *et al*. [27] performed a nearly identical experiment using radiotelemetry instead of tail-cuff plethysmography, no increase in basal blood pressure were observed in the offspring, although they did have an exaggerated pressor response to a stressor. This suggests elevations in blood pressure following DEX exposure may only be apparent in slightly “stressed” animals, which may be due in part to alterations in the hypothalamic-pituitary-adrenal axis (HPA). Certainly, in studies in the maternal protein restriction rat model it has been shown, when telemetry was used to measure blood pressure, that there were no differences in blood pressure in the intrauterine-growth restricted offspring but the offspring did demonstrate an elevated blood pressure in response to a restraint stress [28], [29]. This could account for the reported elevations in blood pressure in this model when blood pressure was measured using the tail-cuff method in unconditioned restrained rats.

Despite good evidence that DEX impairs renal development [30], no studies have examined whether prenatal DEX exposure affects cardiomyocyte number. The major aim of this study was thus to examine the effects of short-term, mid-gestation prenatal glucocorticoid exposure on cardiomyocyte number and cardiac growth factor expression. In addition, we aimed to examine if maternal DEX exposure caused a similar reduction in nephron endowment in mice as observed in other species, and whether this was associated with changes in blood pressure in male offspring. The period of DEX administration in this study, from embryonic day (E) 12.5 to E15, represents critical periods in kidney and heart development in the mouse. In particular, branching morphogenesis in the kidney occurs during this period [31], and myocardial volume doubles [32]. We hypothesized that DEX exposure during development will lead to a reduced cardiomyocyte and nephron endowment leading to long-term cardiovascular disease outcomes, such as hypertension. Finally, we tested whether a stress challenge would exacerbate this phenotype.

## Materials and Methods

### Animals

All experiments were approved in advance by The University of Queensland Animal Ethics Committee and carried out in accordance with the Australian Code of Practice for the Care and Use of Animals for Scientific Purposes. Nulliparous C57BL/6 mice were time mated over a 3 h period. Pregnancy was confirmed by the presence of seminal plugs and this time was recorded embryonic day (E) 0.5. All mice were individually housed in standard rodent cages with access to food and water *ad libitum*. A 12 h light/dark cycle was maintained (0600–1800 h respectively). Pregnant mice underwent surgery at E12.5 for the implantation of a miniature osmotic pump as described previously [20]. The osmotic pumps were filled with either DEX (DEX sodium phosphate, Intervet, Australia; 1 µg/kg/h) or isotonic saline (SAL).

### Tissue collection

Dams were euthanised at E14.5 after 48 h of DEX infusion, or at E17.5, approximately 60 h after DEX infusion had finished (N = 7–8). Fetuses were removed and weighed before their hearts and kidneys were dissected, weighed and snap frozen in liquid nitrogen. A subset of pregnant dams was allowed to litter down. Their offspring were weighed regularly and kept to approximately 12 months of age for blood pressure radiotelemetry measurements, assessment of cardiomyocyte number and nephron number.

### Gene expression

Total RNA was extracted (RNeasy micro-kit QIAGEN, Australia) from whole fetal heart and kidneys. The Applied Biosystems TaqMan Reverse Transcription reagents kit was used to convert 1 µg of RNA into cDNA for real-time PCR. Real-time PCR was performed using 20 ng of cDNA per reaction on a StepOne Real-Time PCR System (Applied Biosystems). The mRNA levels of genes of the renin-angiotensin system (RAS), AT_1a_R and AT_1b_R; canonical cardiac growth factors, insulin-like growth factor 1 (IGF-1), fibroblast growth factor 2 (FGF-2), and vascular endothelial growth factor a (VEGFa); the apoptotic remodeling genes B-cell lymphoma 2 (Bcl-2) and Bcl-2-associated X protein (Bax); genes involved in the development of cardiac contractile function, alpha myosin heavy chain (MHC-α), cardiac sarco/endoplasmic reticulum Ca^2+^-ATPase (SERCA2), and the cardiac ryanodine receptor 2 (Ryr2); as well as glucocorticoid inducible genes, serum glucocorticoid kinase-1 (SGK1) and the glucocorticoid receptor (GR) were measured. Custom probes and primers to detect AT_1a_R and AT_1b_R mRNA levels were used as previously described [7]. TaqMan Assay on Demand assays (Applied Biosystems) were used for IGF-1 (Mm00439560_m1), FGF-2 (Mm00433287_m1), VEGFa (Mm00437304_m1), Bcl-2 (Mm00437783_m1), Bax (Mm00432051_m1), MHC-α (Mm00440359_m1), SERCA2a (Mm01201431_m1), Ryr2 (Mm00465877_m1), SGK1 (Mm00441387_g1) and GR (Mm00433832_m1). The comparative cycle threshold (C_T_) method was used for all expression assays which were run in multiplex reactions with ribosomal 18s RNA used as an endogenous control. The sex of the fetus was determined by the expression level of the sex-specific Xist (Mm01232884_m1) gene.

### Blood pressure measurement

The aged male offspring were placed under general anesthesia (Isoflurane; 3% in 100% oxygen, 125 ml/min) for implantation of radiotelemetry transmitters as previously described [33]. Each animal was allowed 10 days to ensure recovery of normal circadian patterns before measurements commenced. Systolic blood pressure (SBP), diastolic blood pressure (DBP) and activity were measured and heart rate (HR), pulse pressure (PP) and mean arterial pressure (MAP) calculated from these parameters. The probe sampled these measurements at a rate of 10 seconds every 15 min for 7 d. All measurements recorded in each 12 h day/night cycle were averaged to obtain a single value for each period.

### Restraint stress

A baseline measure was obtained from data sampled for 10 seconds, every 5 min, for the hour immediately before the animal was placed in the restraint tube. The animals were placed in a clear, perspex cylinder, just slightly larger than the animal itself (approximately 8 cm long by 4 cm diameter) for 15 min. The animal was then released back into its cage. During the restraint and the subsequent 15 min recovery period data was sampled at a rate of 10 seconds every minute.

### Tissue preparation

At completion of all experiments, mice were euthanized by carbon dioxide. The hearts and kidneys from the aged offspring were removed, weighed and immersion fixed in 4% paraformaldehyde. Whole fixed right kidneys from aged male mice were processed to paraffin wax before being exhaustively sectioned at 5 µm. 10 section pairs, 100 sections apart, were collected to determine glomerular number using unbiased stereology.

### Glomerular number

Glomerular number was determined using the physical dissector-fractionator, as previously reported [34].

### Cardiomyocyte number

The fixed hearts excised from the 12 month old offspring were systematically sampled and embedded in glycolmethacrylate. Using 20 μm glycolmethacrylate sections, an optical disector/fractionator approach was used to determine the total number of cardiomyocyte nuclei within the heart. To do this, using an unbiased counting frame cardiomyocyte nuclei were counted in a systematic uniform sample of fields. The total number of cardiomyocyte nuclei within the heart was then determined by multiplying the number of cardiomyocyte nuclei counted by the reciprocal of the sampling fractions. This method for counting cardiomyocytes is described in detail previously [3]. Since all cardiomyocytes are binucleated in the adult mouse heart (which was visually confirmed), the number of nuclei counted was divided by a factor of 2 to derive the total cardiomyocyte number.

### Statistics

Values are reported as mean ± standard error of the mean. Fetal weights, offspring weights and organ weights were analysed as litter averages. Two-tailed, unpaired Student's *t-tests* were performed to compare between the mean values of the SAL and DEX groups. A multivariate analysis of variance (MANOVA) was used to examine differences between the blood pressure parameters of SAL and DEX exposed males. Prenatal treatment and light/dark periods were entered as dependent variables and litter identification number and time of day assigned as random variables. Statistical significance was defined as *P*<0.05.

## Results

### Maternal characteristics

**Figure 1:**
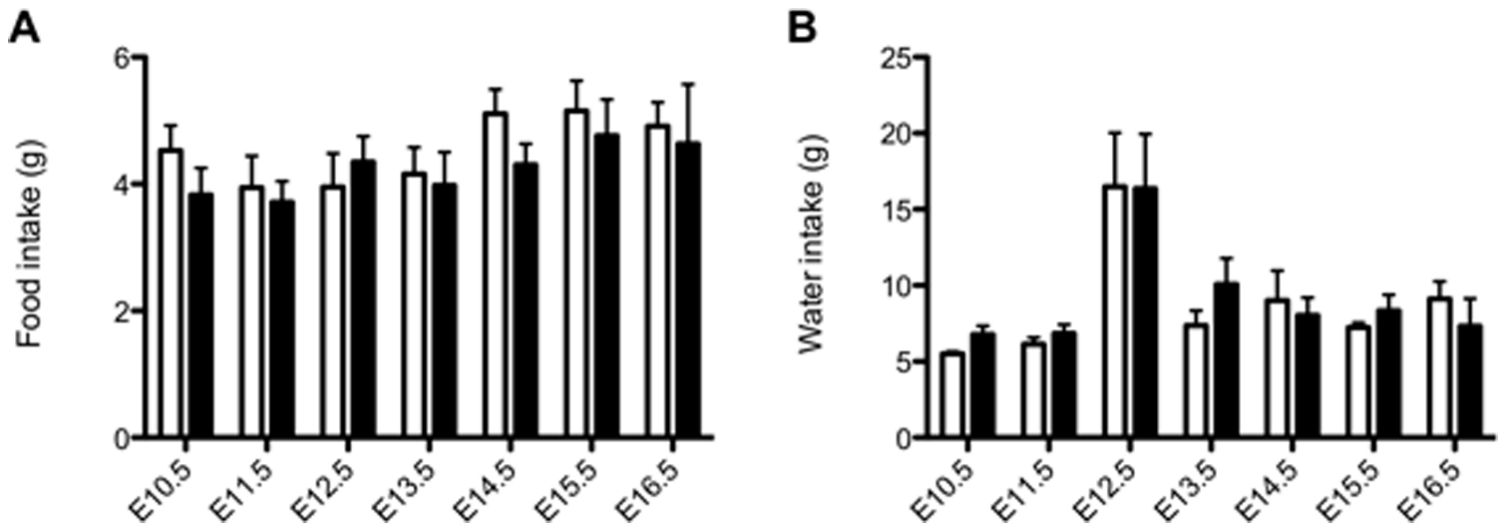
Maternal food and water consumption during pregnancy. The food intake (A) and water intake (B) of the pregnant dams was measured daily from E10.5 to E16.5. The infusion of SAL (open bars) and DEX (closed bars) was for 60 h starting from E12.5. Data is presented as presented as mean ± SEM. N = 5–10 dams per group per day.

There was no difference in body weight at the start of pregnancy (E0) between groups. Both SAL and DEX treated dams also gained weight at similar rate throughout the treatment period (data not shown). Daily food and water intake was not different between groups between E10.5 and E16.5. Water consumption increased in the 24 h following the implantation of the miniature osmotic pump, but this was similar in both SAL and DEX exposed dams (Fig. 1). Litter size did not differ between groups at E14.5 (SAL 7.9±0.7 vs. DEX 7.3±0.6 fetuses) or E17.5 (8.1±0.5 vs. 8.3±0.1 fetuses). Litter size at PN2 was also not different (6.7±0.9 vs. 7.8±0.7 pups).

### Fetal weights

**Figure 2:**
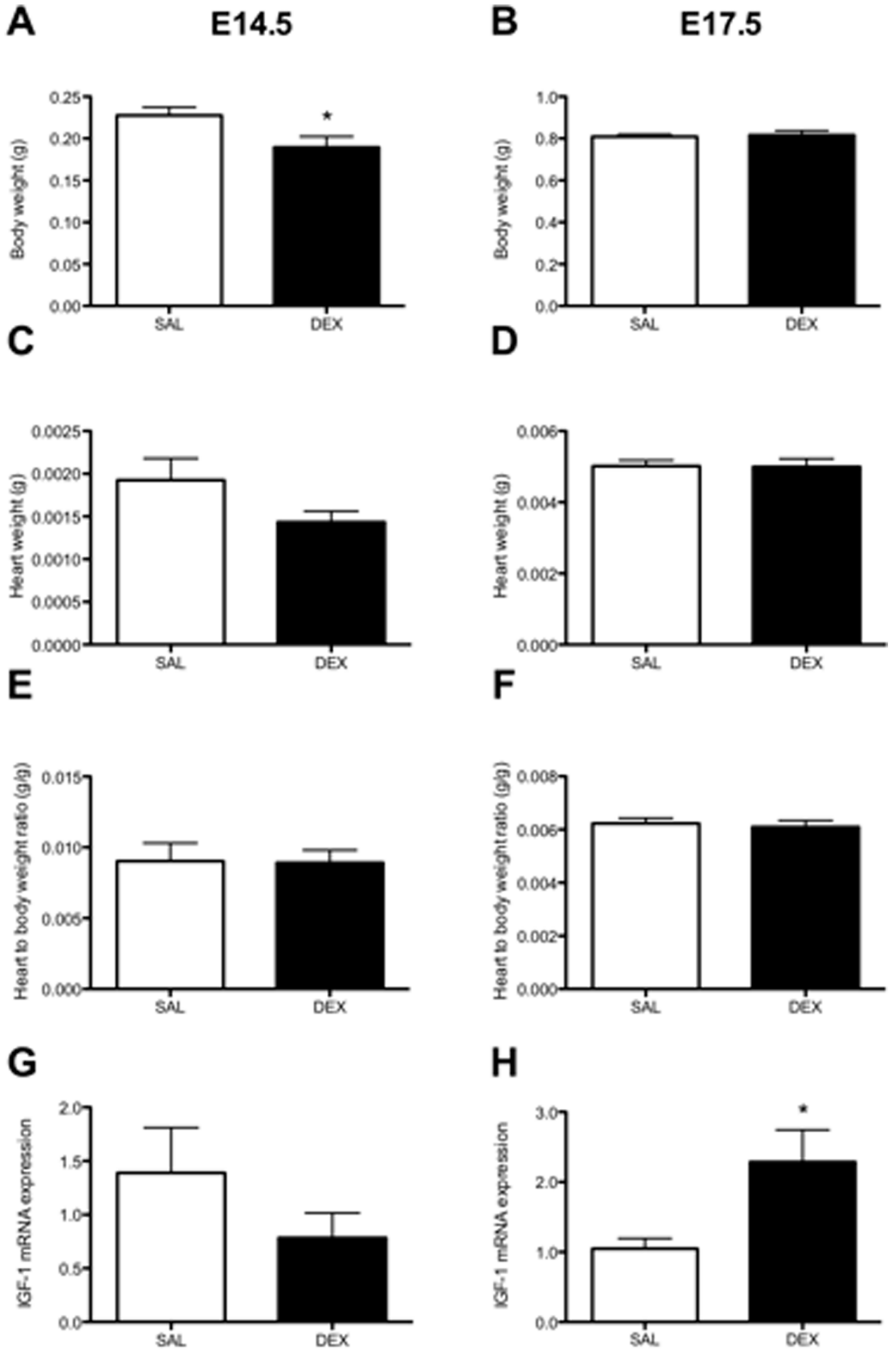
Fetal body weight, heart weight, heart to body weight ratio and IGF-1 mRNA expression. The body weights (A and B), heart weights (C and D) and heart to body weight ratios (E and F) of male fetuses as measured at post mortem tissue collection at embryonic day (E) 14.5 or E17.5. The mRNA levels of insulin like growth factor 1 (IGF-1) at E14.5 (G) and E17.5 (H) are shown, as measured by real-time PCR using the comparative cycle threshold method. Data is presented as litter mean of sexed fetuses ± SEM. N = 7–8 litters (one animal per litter). * *P*<0.05 unpaired Student's *t*-test.

Body weights of the male fetuses at E14.5 were significantly lower in the DEX group compared to SAL (*P*<0.05, Fig. 2A) but were similar to the SAL fetuses by E17.5 (Fig. 2B). Heart weight at E14.5 tended to be smaller in the DEX exposed fetuses (*P* = 0.07, Fig. 2C), but there was no difference in the heart to body weight ratio between the groups (Fig. 2E). The heart weight at E17.5 showed no difference between groups (Fig. 2D) and the heart to body weight ratio were not different (Fig. 2F). Likewise, heart volume at E17.5 was not different between groups (SAL 107.7±5.5 mm^3^ vs. DEX 113.5±4.2 mm^3^) and the heart volume to body weight ratio (SAL 2.9±0.2 mm^3^/g vs. DEX 2.8±0.2 mm^3^/g) were unchanged (data not shown). Kidneys were not weighed at E14.5 as their very small size made accurate weighing difficult. However, there were no differences in kidney weight (SAL 6.6±0.3 mg vs. DEX 7.2±0.5 mg) or kidney weight to body weight ratio (SAL 8.2±0.3 mg/g vs. DEX 8.8±0.5 mg/g) at E17.5.

### Cardiac mRNA expression

At E14.5 there were no significant differences in the mRNA levels of any of the genes examined (Table 1). At E17.5 there were significantly higher mRNA levels of AT_1a_R and Bax in the DEX group compared to SAL (Table 1). There was also a significant increase in IGF-1 mRNA levels at E17.5 in the DEX group (*P*<0.05, Fig. 2H). DEX did tend to increase expression of both the GR and SGK1 at E14.5 and E17.5, although this was only significant for the GR at E17.5. All other genes examined at E17.5 showed no significant differences between groups.

**Table 1:** Cardiac mRNA levels of genes involved in heart growth and apoptosis at E14.5 and E17.5 in male fetuses prenatally exposed to saline (SAL) or dexamethasone (DEX).

	E14.5		E17.5	
*RAS genes*	SAL	DEX	SAL	DEX
**AT_1a_R**	1.07±0.17	1.10±0.22	1.05±0.14	2.64±0.38*
**AT_1b_R**	1.16±0.27	0.88±0.17	1.16±0.36	0.88±0.32
***Cardiac growth factor genes***				
**IGF-2**	1.04±0.12	1.04±0.26	1.44±0.46	1.13±0.48
**FGF-2**	1.13±0.20	0.74±0.09	1.08±0.16	0.92±0.11
**VEGFa**	1.04±0.11	1.23±0.18	1.01±0.09	0.85±0.12
***Apoptotic genes***				
**Bax**	1.01±0.04	0.96±0.10	0.96±0.04	1.93±0.20*
**Bcl-2**	1.06±0.13	1.28±0.12	1.21±0.29	1.01±0.58
***Contractile function genes***				
**MHC-α**	0.98±0.30	0.47±0.16	1.14±0.39	1.27±0.32
**SERCA2**	1.18±0.38	0.73±0.16	1.08±0.19	1.46±0.24
**Ryr2**	0.95±0.30	0.66±0.27	1.25±0.45	1.56±0.32
***Glucocorticoid regulated genes***				
**GR**	1.08±0.16	2.42±0.79	1.02±0.10	3.04±0.47*
**SGK1**	1.19±0.57	2.67±0.85	1.11±0.42	1.97±0.40

Values are presented relative to SAL at for each age using comparative cycle threshold (C_T_) method. Data presented as the mean ± SEM. N = 5–9 litters (one animal per litter) per group at each age.* *P*<0.05 unpaired Student's *t*-test SAL *vs.* DEX.

### Postnatal growth

There were no differences in body weights between the SAL and DEX exposed male offspring at 2 weeks, 4 weeks, 3 months and 6 months of age (Table 2).

**Table 2:** Postnatal body weights of male offspring prenatally exposed to saline (SAL) or dexamethasone (DEX).

	Body weight (g)			
	2 weeks	4 weeks	3 months	6 months
**SAL**	6.4±0.9	12.1±0.9	28.3±1.0	35.6±1.3
**DEX**	5.7±0.2	11.9±0.2	28.3±0.4	33.2±1.1

Data presents as means ± SEM, N = 6–8 litters (one animal per litter) per group.

### Basal blood pressure

**Figure 3:**
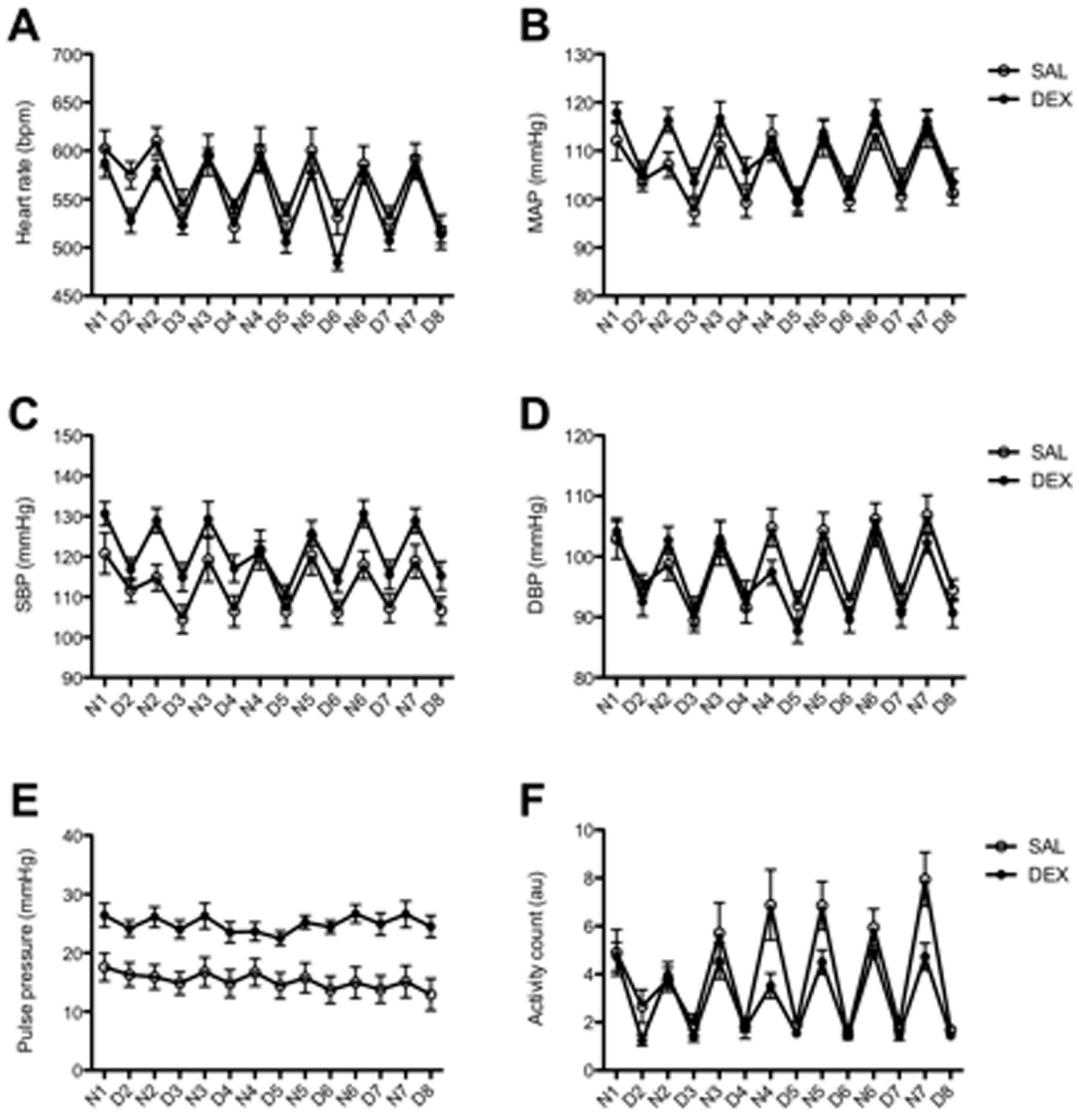
Basal heart rate, mean arterial pressure and the pulse pressure in aged male offspring. The basal heart rate, mean arterial pressure (MAP) and pulse pressure of mice prenatally exposed to SAL (open circles) or DEX (closed circles) as measured by radio telemetry. Each data point represents the mean of the sampled data collected for 10 seconds every 15 minutes for each 12 h night (N) and day (D) cycle. Measurements were started after 10 days post surgical implantation of the radio transmitter to allow the mice to recover normal circadian variations. Data is presented as mean ± SEM. N = 6–7 litters (one animal per litter). * *P*<0.05 by Repeated measures one way ANOVA.

Significant effects of prenatal DEX were observed with increases in basal HR, MAP (∼3 mmHg), SBP, PP and activity in exposed offspring at 12 months of age (Tables 3–4). Normal circadian rhythm was observed across all of these parameters, including nocturnal dipping. DEX exposed male offspring were also significantly less active during their more active dark period (Fig. 3F).

**Table 3:** Mean values of basal blood pressure telemetry parameters for the light and dark periods and overall daily value for 12 month old male offspring of DEX and SAL exposed dams.

		HR (bpm)	MAP (mmHg)	SBP (mmHg)	DBP (mmHg)	PP (mmHg)	Activity (a.u.)
**SAL**		**Day**	534.8±40.7	100.2±6.2	107.0±8.1	92.6±4.9	14.4±5.3
	**Night**	598.1±45.0	112.1±8.8	119.1±10.5	103.7±7.5	16.1±5.6	1.9±0.9
	**Total**	566.5±53.2	106.2±9.6	113.0±11.1	98.2±8.4	15.2±5.5	6.0±2.7
**DEX**	**Day**	514.8±29.6	103.4±6.8	114.8±8.3	90.8±6.0	24.0±3.9	4.0±2.9
	**Night**	514.8±29.6	115.5±6.9	127.9±8.7	102.0±6.7	25.8±4.6	1.5±0.6
	**Total**	549.5±45.4	109.4±9.1	121.4±10.7	97.2±8.3	24.9±4.4	4.4±1.4

Data presented as mean ± standard deviation for all data collected over 7 day period. HR, heart rate; MAP, mean arterial pressure; SBP, systolic blood pressure; DBP, diastolic blood pressure; PP, pulse pressure.

**Table 4:** *P* values for differences in basal blood pressure parameters as calculated by MANOVA.

	HR (bpm)	MAP (mmHg)	SBP (mmHg)	DBP (mmHg)	PP (mmHg)	Activity (a.u.)
**Treatment**	**0.002**	**0.003**	**0.001**	**0.052**	**0.001**	**0.001**
**Period**	**0.001**	**0.001**	**0.001**	**0.001**	**0.014**	**0.001**
**Treatment × Period**	0.567	0.908	0.700	0.954	0.925	**0.015**

Bold numbers indicate significant (*P*<0.05) effect of treatment, period or a treatment by period interaction. HR, heart rate; MAP, mean arterial pressure; SBP, systolic blood pressure; DBP, diastolic blood pressure; PP, pulse pressure.

### Blood pressure response during restraint stress

**Figure 4:**
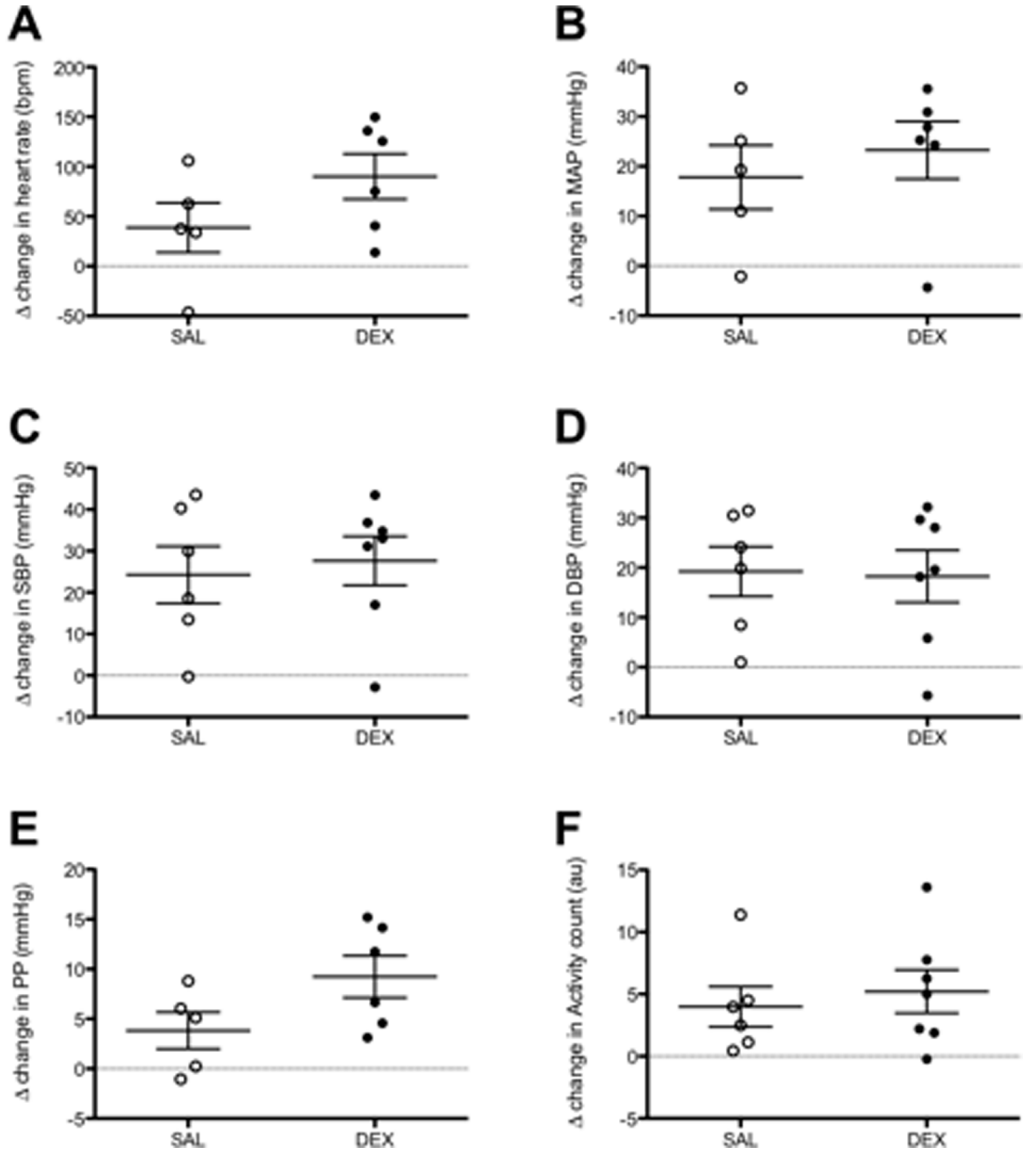
Heart rate, mean arterial pressure and pulse pressure responses to restraint stress. The delta change heart rate (A), mean arterial pressure (MAP; B) and pulse pressure (PP; C) after exposing the aged male mice to a 15 minute restraint stress from baseline values. The data points represent the Δ value between the mean of the data sampled during the restraint stress (10 seconds every minute for 15 minutes) from the baseline value (mean of data sampled 10 seconds every 5 minutes in the hour immediately prior to the restraint stress). SAL (open circles), DEX (closed circles). Data presented as the mean ± SEM. N = 6–7 litters (one animal per litter).

No significant changes in HR, MAP, SBP, or DBP responses to restraint stress were observed in 12-month-old offspring (Fig. 4A–D). There was a tendency for DEX exposed males to have an increased widening of the pulse pressure during the restraint stress (*P* = 0.09, Fig. 4E).

### Post mortem body, heart and kidney weights at 12 months of age

**Figure 5:**
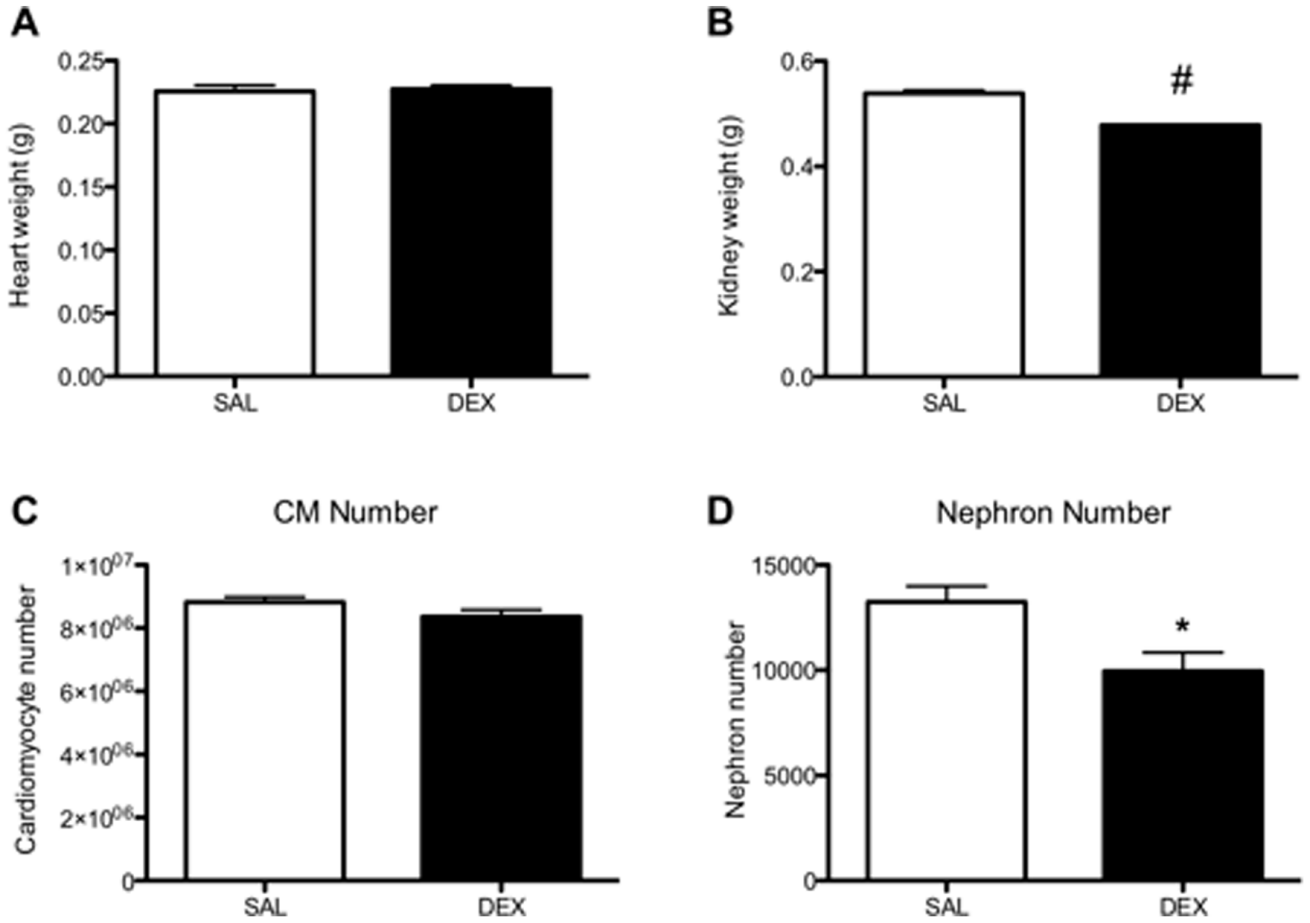
Effects of dexamethasone exposure on nephron number and cardiomyocyte number. The number of nephrons in the kidney (A), and the number of cardiomyocytes (B) in the heart from aged male mice were assessed by unbiased stereology. Open bar indicates SAL exposed animals and the closed bar DEX exposed animals. Data is presented as mean ± SEM. N = 6 kidneys per group and N = 5 hearts per group (one animal per litter). * *P*<0.05, # *P*<0.0001 by unpaired Student's *t*-test.

At the time of post mortem tissue collection there was no difference in body weight between SAL and DEX exposed offspring (38.9±1.1 g vs. 37.4±0.2 g, respectively). Heart weight (Fig. 5A) and heart to body weight ratio were not different between groups. The DEX exposed offspring had a significantly lower total kidney weight (*P*<0.01, Fig. 5B) and a significantly lower kidney to body weight ratio (P<0.001, data not shown).

### Cardiomyocyte and nephron number at 12 months of age

The number of cardiomyocytes in the adult male hearts was not significantly different between the SAL and DEX groups (Fig. 5C). DEX exposed males had a significant nephron deficit of approximately 25% compared to SAL exposed males at 12 months of age (Fig. 5D).

## Discussion

As we have reported previously, DEX exposed male fetuses had a transient growth restriction [20]. They also had a tendency to have smaller hearts at E14.5, but approximately 2 days after the DEX infusion had ceased both body weight and heart weight were similar to control suggesting accelerated growth between E15 and E17.5. This catch-up in heart growth is associated with increased cardiac mRNA expression of the AT_1a_R, Bax and IGF-1 genes. In adult life, the DEX exposed males did not develop a hypertensive phenotype, and had a similar blood pressure response to an acute stress as their SAL exposed counterparts, despite having fewer nephrons. DEX exposed males did have a widened pulse pressure in later life compared to controls which may suggest an alteration in the vascular compliance of these mice.

### Growth restriction

Growth restriction is a common finding in models of long-term glucocorticoid exposure [35], [36], whereas short-term exposure to glucocorticoids does not usually result in restricted growth [37], [38], [39], [40], [41], [42]. In the present study, however, 48 h of DEX exposure, did lead to significantly reduced body size in the period during the DEX exposure at E14.5, but body size was restored to normal by E17.5 and there was no evidence of postnatal growth restriction. Experimental differences such as: the timing of glucocorticoid administration during gestation, dosage, duration of exposure, and animal species used, may all contribute to the variations seen in glucocorticoid induced growth restriction. In contrast to rat studies in which glucocorticoids have been administered during pregnancy, our model did not induce hypophagia in the pregnant dams [16], [43]. This finding strongly suggests that the transient growth restriction is a result of a direct effect of the DEX on fetal growth pathways, and not due to fetal under nutrition as a result of reduced maternal food intake. Our model is not confounded by low birth weight, a common finding of many other models of glucocorticoid induced fetal programming, also our DEX exposed male offspring were not growth restricted. Our brief mid-gestational period of maternal DEX exposure may account for these differences.

### Cardiac growth and gene expression

The transient growth restriction was also reflected in a tendency (*P* = 0.07) for the heart weights of the DEX fetuses to be lower at E14.5; this did not quite reach statistical significance probably due to the inaccuracies in weighing hearts of such small size. Overall, our findings suggest that the reduction in cardiac growth was a direct consequence of the fetal growth restriction, with no difference in the heart weight to body weight ratio between groups. By E17.5 there were no differences between body weights or heart weight (both absolute and normalized to body weight) between groups implying that there was compensatory ‘catch up’ in cardiac and body growth after the cessation of DEX exposure. This compensatory growth did not alter the mRNA expression of key genes related to cardiac contractility. Few studies have examined the direct effects of DEX on heart weight or in the immediate aftermath of exposure with the focus generally on body and organ weights at the birth, or in the neonatal period. Ovine fetal heart weight following prolonged exposure to a low dose of DEX (20 µg/g/d from 25–45 d) showed no difference at day 45 of gestation but a reduction in heart weight was observed at day 130 (term 150 d) [41].

To investigate which molecular signals may contribute to the slowed heart growth at E14.5, and the ‘catch-up’ growth of the heart at E17.5 we examined the mRNA expression levels of the RAS genes, canonical cardiac growth factors, and apoptotic genes that play a crucial role in cardiac growth and remodeling. Interestingly, none of the genes we examined at E14.5 showed any significant difference in expression in the DEX exposed fetuses compared to controls. As these hearts were examined during the DEX infusion, this suggests that there was no direct effect of the DEX on the cardiac expression of these genes. However, it is to be noted that there was an approximate 45% reduction in the IGF-1 mRNA levels (although not statistically significant due to the wide variation within groups), which may have contributed to the slowing of cardiac growth. The lack of gene expression changes within the heart is somewhat surprising given we have previously shown that DEX exposure has caused changes in mRNA levels genes such as the VEGFa receptor KDR in the placenta in this model [20]. Also, in the hearts of growth restricted E12.5 mouse fetuses (induced by 6 h of 8% oxygen prior to E12.5) VEGFa mRNA was increased 1.9 fold, but no changes were reported in other growth factors measured such as FGF-2 mRNA [44]. Although the expression of the genes selected in this study were not changed during DEX exposure, it is likely that other relevant genes may have been affected such as the recently identified novel cardiotropic factor integrin-linked kinase [45].

At E17.5 the increased mRNA levels of cardiac AT_1a_R and IGF-1 in the DEX exposed fetuses would suggest an up regulation of cardiac growth pathways, which likely contributed to the restoration of heart weight by this time. The AT_1a_R receptor is the predominant angiotensin II receptor subtype in the heart [46] and it plays a key role in mediating the myocardial trophic effects of angiotensin II [47], [48] stimulating both hypertrophy and/or hyperplasia. IGF-1 plays a major role in cardiac growth, stimulating cardiomyocyte hypertrophy and hyperplasia. The immature mouse cardiomyocytes are capable of both proliferation and hypertrophy at E17.5 [48], [49]. Hence, it is likely that the increase in AT_1a_R and IGF-1 mRNA levels in DEX exposed fetuses at E17.5 may be driving the increase in heart weight from its relatively small size at E14.5 back to normal by E17.5. The up-regulation of Bax mRNA expression that we observed at E17.5 suggests that the apoptotic process has seen an increase in activation. Importantly, the increase in the pro-apoptotic Bax gene in the myocardium at E17.5 suggests that exposure to DEX may have led to increased apoptosis of cardiomyocytes, and thus may have contributed to the reduced cardiac size in the DEX-exposed fetuses; whether this was a generalized phenomenon in other tissues was not examined. Indeed, it may be the increase in cardiomyocyte apoptosis following DEX-exposure that that has led to a reactive rise in IGF-1. We have previously shown a reactive rise in cardiac mRNA IGF-1 levels following induction of apoptotic genes in the fetal sheep heart when it was exposed acutely in late-gestation to an insult of maternal alcohol consumption [50]. Having established a model of prenatal glucocorticoid fetal programming in the mouse we hope it will be possible to now utilize some molecular and genomic tools optimized for the mouse, such as gene microarray technologies, to explore in more detail the underlying mechanistic causes to the cardiovascular and renal phenotype in this model.

Our findings (on body and heart weights and gene expression at E17.5) imply that the developing fetal mouse heart, although adversely affected by 60 hours DEX-exposure from E12.5, was able to undergo subsequent compensatory growth *in utero*, such that heart growth at birth, and postnatally, was not adversely affected. No difference in total cardiomyocyte number in the DEX-exposed and control hearts at 12 months of age supports this. Importantly, in this regard, proliferation of cardiomyocytes predominantly ceases soon after birth and so the observed cardiomyocyte number in the adult offspring likely reflects cardiomyocyte endowment at the beginning of life, since there were no differences between the experimental groups, or in the way the offspring were treated postnatally.

### Basal blood pressure

In our model of short-term, mid-gestation DEX exposure there was evidence of a subtle, but statistically significant increase in blood pressure in the male offspring at 12 months of age. The increase in SBP of ∼8 mmHg in the DEX exposed offspring was the most pronounced alteration and was consistent over the 7 day data collection period (Fig. 3C). During the dark period, when the DEX exposed males were less active, the increase in SBP became most notable. However, the magnitude of the HR, MAP and DBP increases in the DEX exposed group are smaller, and in most instances not larger than the stated accuracy of the telemetry transmitter (±3 mmHg) suggesting that DEX has not programmed overt hypertension. The development of hypertension in adult life after prenatal exposure to glucocorticoids is a common finding [18], [26], [36], [43], [51], although this is not always the case [27], [52], [53]. In this regard, it is important to note that the use of methods such as tail-cuff plethysmography or carotid cannulation to measure blood pressure, may elicit a stress response and thus not truly reflect elevations in basal blood pressure [28], though repeated handling and conditioning of the animal through multiple, repeated measurements made by experienced experimenters may minimize the effects of these stresses and still yield valuable data. Other factors such as recovery from anesthesia, or preheating of the tail have the potential to lead to physiological alterations in the animal's cardiovascular system and a subsequent a rise in blood pressure. Indeed, in a number of studies (in sheep, monkeys and rats) where blood pressure was measured using non-invasive radiotelemetry there was no evidence of a hypertensive phenotype in the offspring that had been parentally exposed to DEX [27], [52], [53]. Alternatively, O'Regan *et al.* reported that their DEX exposed rat offspring were hypotensive relative to the control group, that the male offspring also had an exaggerated blood pressure response to a restraint stress, and were less active [27]. Other studies in rats have also shown an elevated blood pressure response to stress in offspring that have been exposed to excess levels of glucocorticoids, but without basal hypertension [54], [55]. These differences may be due to the differences in the timing of glucocorticoid exposure between our studies and those mentioned above. Importantly, in relation to this, HPA development and maturation is highly species specific, and in the rodent much of this maturation occurs in the early postnatal period [56]. The HPA axis is altered by prenatal exposure to glucocorticoids, and some of those alterations persist into adulthood [18], [57]. For example, in studies where rat dams were exposed to restraint stress during the last week of pregnancy [54], [55], the offspring had an elevated blood pressure response to stressors. This window of prenatal exposure is a lot closer to the critical window of HPA axis maturation than the period of exposure used in this study (60 h from E12.5). It is possible that the length of exposure and relatively early timing of DEX administration in our model did not sufficiently program the HPA axis to elicit a significant increase in blood pressure in response to stress.

The widened pulse pressure observed in the DEX exposed male offspring was an unexpected finding and may indicate alterations in vascular compliance. Changes in the vasculature of prenatally insulted offspring have been reported widely in the literature [13], [58], [59]. In particular, aged male rats that were subjected to intrauterine growth restriction had a widened pulse pressure at 9 months, and an even wider pulse pressure at 12 months of age relative to controls [59]. Stiffening of the arteries in offspring following maternal high fat feeding uteroplacental insufficiency has also been reported [13], [60]. Hence, arterial stiffness may be contributing to the widened pulse pressure reported in this study and merits further investigation into the composition and function of the vessels from similarly treated animals.

### Nephron number

We report for the first time that prenatal DEX exposure in the mouse results in a significant reduction in nephron number in the offspring. This finding is in agreement with similar findings across a wide range of other species following prenatal DEX exposure such as sheep, rats and spiny mice [21], [22], [51]. The nephron deficit of around 25% in our aged male offspring was not associated with an elevation in blood pressure. Although a reduction in nephron endowment leads to vulnerability to the development of hypertension, our findings are in accordance with many other studies, which show that an elevation in blood pressure is not a direct corollary of a reduced nephron endowment [9], [22], [61], [62]. Although kidney function was not measured in this study we observed no overt signs of renal pathology in the histological sections used for nephron number counting. This is not surprising given that the C57BL/6 mouse strain is relatively resistant to developing glomerulosclerosis following a reduction in renal mass [63]. Whether an additional insult to the renal system would lead to an overt pathological phenotype is yet to be elucidated.

### Conclusion

In conclusion, the findings of this study suggest that short-term exposure to DEX in mid-gestation adversely impacts on nephrogenesis and fetal cardiac growth. Encouragingly, the developing heart appears to be able to compensate, by accelerated growth after withdrawal of the insult, such that cardiomyocyte endowment and postnatal cardiac growth are not affected. Although nephron endowment is reduced, there is only a small increase in SBP in adulthood, and no difference in the blood pressure response to stress. A widening of the pulse pressure seen in DEX-exposed offspring may be indicative of programming changes to vascular compliance and this warrants further investigation.
